# A CTS Team Approach to Wastewater-Based Epidemiology of Non-Typhoidal Salmonella in Gainesville, FL – ERRATUM

**DOI:** 10.1017/cts.2022.416

**Published:** 2022-09-02

**Authors:** Andrew Rainey, Lisa E. Emerson, Mariola J. Edelmann, Anthony T. Maurelli

The above abstract [1] published without listing Lisa E. Emerson as an author.

The original article has been corrected online to rectify this error. The editors apologize for this oversight.
